# Severe Acute Respiratory Infection (SARI) due to Influenza in Post‐COVID Resurgence: Disproportionate Impact on Older Māori and Pacific Peoples

**DOI:** 10.1111/irv.70029

**Published:** 2024-10-30

**Authors:** Isabella M. Y. Cheung, Janine Paynter, David Broderick, Adrian Trenholme, Cass A. Byrnes, Cameron C. Grant, S. Qiu Huang, Nikki Turner, Peter McIntyre

**Affiliations:** ^1^ Department of General Practice and Primary Health Care University of Auckland Auckland New Zealand; ^2^ Department of Paediatrics: Child and Youth Health University of Auckland Auckland New Zealand; ^3^ Starship Children's Hospital, Te Whatu Ora – Health New Zealand Te Toka Tumai Auckland Auckland New Zealand; ^4^ Institute of Environmental Science and Research Wellington New Zealand; ^5^ Department of Women's and Children's Health University of Otago Dunedin New Zealand

**Keywords:** influenza, equity, post‐covid lockdowns

## Abstract

**Objective:**

Influenza reemerged after a 2020–2021 hiatus in 2022, but understanding the resurgence needs pre‐COVID era surveillance. We compared age‐ and ethnicity‐specific incidence of severe acute respiratory infection (SARI) from a hospital network in Auckland, New Zealand, in 2022 against a baseline, 2012–2019.

**Methods:**

Annual and monthly influenza SARI incidence per 1000 persons by age and ethnic group between 2012 and 2022 was calculated using resident population as the denominator. The hospitals capture most severe illness of the resident population.

**Results:**

Influenza SARI incidence was highest among <1 year olds (2.62; 95% CI: 1.84–3.61) during 2012–2019, lowest at 6–14 years, and did not significantly increase until 50–64 years (0.35; 95% CI: 0.27–0.45), reaching 1.19 (95% CI: 0.57–1.55) in those ≥75 years. In all age groups, incidence was at least threefold higher in Māori and Pacific Peoples. No influenza SARI was identified in 2020–2021. In 2022, despite an early peak, annual incidence (<65 years) was lower than baseline in all ethnic groups, but incidence (≥65 years) in Māori (2.06; 95% CI: 1.22–3.26) and Pacific (3.94; 95% CI: 2.97–5.13) peoples was higher in 2022 than most baseline years, whereas incidence in NMNP (0.22; 95% CI: 0.14–0.32) was lower than any baseline year.

**Conclusion:**

After no influenza 2020–2021, Auckland had an early, high, narrow peak in 2022. Stratification by age and ethnicity revealed striking discrepancies in incidence among Māori and Pacific adults over 65 years compared with NMNP adults, with implications for targeted vaccination strategies.

## 1 Introduction

Surveillance using the severe acute respiratory infection (SARI) case definition of hospitalization with fever, and cough of fewer than 10 days' duration, allows an internationally applicable approach to tracking influenza activity. This was implemented by the World Health Organization (WHO) following the 2009 influenza pandemic [1].

In 2012, New Zealand (NZ) established population‐based influenza surveillance, the Southern Hemisphere Influenza and Vaccine Effectiveness Research and Surveillance (SHIVERS) project [2]. Surveillance included daily ascertainment of cases meeting the SARI definition through a hospital network in the largest city (Auckland), with polymerase chain reaction (PCR)‐based testing for influenza and other respiratory viruses. SHIVERS has delivered stable surveillance of influenza‐associated SARI for one of the longest periods worldwide [3].

On 11 March 2020, WHO categorized coronavirus disease (COVID‐19) as a pandemic; 8 days later, NZ closed its international borders and a national lockdown commenced on 25 March 2020. Although large reductions in the transmission of influenza and many other respiratory viruses were observed globally during the COVID‐19 pandemic [3], the island geography of NZ and the extent of border closure likely resulted in very rapid and very large reductions in respiratory viral transmission [4]. SHIVERS SARI surveillance was continuous during the COVID‐19 pandemic, augmented by PCR testing for influenza of persons with acute respiratory symptoms and a negative SARS‐Co‐V2 PCR staying in quarantine facilities. Between April 2020 and the end of 2021, laboratory‐confirmed influenza infection was documented only among people in quarantine facilities [5]. No influenza infections were documented in SARI surveillance sites, despite testing practices remaining unchanged.

In 2022, rebounds in influenza were reported from many countries but were difficult to interpret due to variations in pre‐COVID testing practices and case ascertainment [6]. In NZ, SARI surveillance has been undertaken continuously over a long period using a consistent methodology, allowing SARI‐influenza activity in 2022 to be validly compared with an extended pre‐COVID baseline. This is of importance, given the impact of COVID‐19‐related public health and social measures, and associated perturbations in patterns of diagnostic testing and respiratory viral infection [7, 8].

We recently reported data on the detection of a wide range of respiratory viruses in NZ in 2022, the first year during which international borders were reopened since 2020 [5]. Here, we present a detailed analysis of influenza‐associated SARI by age and ethnic group. This is of importance given previous well‐documented disparities in the burden of respiratory infections [9] and other conditions [10] among Māori and Pacific Peoples (the indigenous peoples of Aotearoa/NZ and the South Pacific islands, respectively). Substantially larger proportions of Māori and Pacific people reside in areas of higher socioeconomic deprivation [11, 12], so ethnicity is likely a proxy for related factors such as housing conditions and crowding [9], access to health services and higher prevalence of comorbidities [13].

We aimed to identify whether the 2022 influenza resurgence had differential impacts by age and ethnicity, likely to be relevant to understanding of influenza activity in other countries in the Western Pacific Region.

## 2 Methods

Population‐based hospital surveillance for SARI included adult and paediatric hospitals in Central and South Auckland, NZ, which serve a well‐defined population of approximately 1 million [14].

SARI cases were ascertained daily, by nurse review of clinical records for acute admissions in the 24 h since last review to assess compliance with the SARI case definition. Inpatients who met the WHO SARI surveillance case definition (history of fever or measured fever of ≥38°C, cough with onset within last 10 days and requiring hospitalization) [1] were enrolled. A nasal, pharyngeal or nasopharyngeal specimen was collected and tested for influenza and other respiratory viruses using real‐time reverse transcription PCR [14].

This analysis included influenza‐positive SARI cases identified from 2012 to 2022. We defined 2012–2019 as pre‐COVID years and 2020 and 2021 as COVID‐19 pandemic years, with international borders opened to vaccinated travellers in February 2022 and to all travellers in May 2022 [5]. Annual and monthly incidence for each year were calculated as the number of cases per 1000 population per year or month, respectively, using population denominators obtained from the 2006, 2013 and 2018 NZ Census from Stats NZ. For intercensus years between 2012 and 2018, population figures were derived using linear interpolation from 2006, 2013 and 2018 Census counts [15]. For intercensus years after 2018, population projections were provided by the NZ Ministry of Health, derived from 2018 Census counts. As testing rates for influenza were relatively high and consistent across most study years (approximately 82%), we did not adjust for SARI cases not tested for influenza in this analysis. The total resident population was assumed to be at risk of influenza.

Annual incidence was based on cases that occurred during the 6 months of peak influenza activity each year (May to October, surveillance weeks 18–43), as active SARI surveillance in sentinel hospitals was limited to this period [14]. Monthly incidence was based on surveillance weeks 18 to 41, to enable aggregation into 4‐week groups; there were no cases in weeks 42 and 43 in any year. Using Byar approximation to Poisson distribution, 95% confidence intervals (CI) for annual incidence were calculated. Due to smaller case numbers, CIs for monthly incidence were calculated using a normal approximation to Poisson distribution. Mean annual incidence of 2012–2019 ± standard deviation (SD) was calculated. Incidence rate ratios (IRR) were also calculated, to compare annual incidence in 2022 with the mean annual incidence of 2012–2019 and where relevant to compare incidence by ethnicity.

Annual and monthly incidence was calculated for the age groups <1, 1–2, 3–5, 6–14, 15–29, 30–49, 50–64, 65–74 and ≥75 years. For annual and monthly incidence by ethnic and age group, age groups were aggregated into ≤14, 15–49, 50–64 and ≥65 years due to smaller case numbers following substratification by age and ethnic group. Incidences were calculated for three ethnic groups: Māori, Pacific and non‐Māori/non‐Pacific (NMNP), which mainly comprised European and Asian ethnic groups.

Ethical approval was granted by the Northern Health and Disability Ethics Committee for deidentified analysis. Study data were collected using REDCap Version 10.0.19 [16]. Data processing and analyses were performed using IBM SPSS Statistics Version 26.

## 3 Results

From 2012 to 2022, 2960 cases of influenza‐associated SARI were identified. There were no cases of influenza‐associated SARI in 2020 or 2021. Influenza A accounted for all cases in 2022 and over 70% of cases in all but two years (2013 and 2015) when Influenza B caused 31% and 37%, respectively (Table 1).

**TABLE 1:** Annual incidence of influenza SARI by age group, Auckland, New Zealand, 2012–2022.

Age group, years	Year									
	2012	2013	2014	2015	2016	2017	2018	2019	2012–2019	2022
All ages	**0.44** **(0.40–0.49)**	0.26 (0.23–0.29)	**0.43** **(0.39–0.48)**	0.35 (0.31–0.39)	0.15 (0.13–0.17)	**0.37** **(0.33–0.41)**	0.26 (0.23–0.29)	0.36 (0.32–0.39)	0.33 (0.29–0.36)	0.29 (0.26–0.33)
<1	**3.95** **(2.99–5.13)**	1.93 (1.28–2.79)	**4.54** **(3.50–5.79)**	**3.11** **(2.26–4.18)**	0.78 (0. 39–1.41)	1.59 (0.99–2.41)	1.90 (1.24–2.79)	**3.01** **(2.17–4.08)**	2.62 (1.84–3.61)	0.91 (0.47–1.59)
1–2	1.48 (1.07–2.00)	0.87 (0.56–1.29)	1.26 (0.88–1.75)	1.31 (0.92–1.81)	0.75 (0.46–1.15)	0.76 (0.47–1.16)	1.09 (0.74–1.56)	1.09 (0.73–1.56)	1.08 (0.73–1.54)	0.65 (0.37–1.04)
3–5	0.36 (0.20–0.59)	0.28 (0.15–0.49)	0.31 (0.16–0.52)	0.35 (0.19–0.58)	0.14 (0.05–0.31)	0.38 (0.22–0.61)	0.28 (0.15–0.50)	0.31 (0.16–0.53)	0.30 (0.15–0.51)	0.37 (0.21–0.61)
6–14	0.14 (0.08–0.22)	0.06 (0.02–0.12)	0.12 (0.06–0.20)	0.09 (0.05–0.16)	0.03 (0.01–0.08)	0.10 (0.05–0.17)	0.10 (0.06–0.18)	0.13 (0.08–0.21)	0.09 (0.04–0.16)	0.16 (0.10–0.24)
15–29	0.20 (0.15–0.27)	0.13 (0.09–0.18)	0.24 (0.18–0.31)	0.16 (0.12–0.22)	0.06 (0.03–0.10)	0.11 (0.08–0.17)	0.08 (0.05–0.12)	0.14 (0.10–0.19)	0.13 (0.09–0.19)	0.18 (0.13–0.24)
30–49	0.26 (0.20–0.33)	0.13 (0.09–0.18)	0.31 (0.25–0.39)	0.19 (0.14–0.25)	0.07 (0.05–0.11)	0.15 (0.11–0.20)	0.15 (0.11–0.20)	0.22 (0.17–0.28)	0.18 (0.13–0.24)	0.19 (0.15–0.25)
50–64	0.46 (0.36–0.58)	0.25 (0.18–0.34)	0.52 (0.42–0.65)	0.38 (0.30–0.49)	0.16 (0.10–0.23)	**0.48** **(0.38–0.59)**	0.32 (0.24–0.41)	0.30 (0.22–0.39)	0.35 (0.27–0.45)	0.28 (0.21–0.37)
65–74	0.80 (0.58–1.06)	0.47 (0.31–0.68)	0.61 (0.43–0.84)	0.55 (0.38–0.76)	0.21 (0.12–0.36)	0.82 (0.62–1.07)	0.54 (0.38–0.75)	0.71 (0.53–0.93)	0.58 (0.41–0.80)	0.66 (0.49–0.86)
≥75	**1.44** **(1.10–1.87)**	1.03 (0.74–1.39)	0.76 (0.52–1.07)	1.14 (0.85–1.51)	0.71 (0.49–1.00)	**2.13** **(1.73–2.59)**	0.77 (0.54–1.07)	**1.49** **(1.18–1.87)**	1.19 (0.57–1.55)	0.80 (0.58–1.07)

*Note:* All values are incidence per 1000 persons (95% confidence interval). No cases of influenza‐associated SARI were detected in 2020 and 2021. Bold values indicate incidence and CI significantly above 2022.

For all ethnic groups combined, annual incidence per 1000 population was highest in infants in all years, pre‐COVID (2012–2019) and post‐COVID (2022) but varied more than fourfold across years and was highest in 2014 and lowest in 2016 (4.55 and 0.79 per 1000 persons, respectively). Annual incidence in 2022 was significantly lower than mean annual incidence 2012–2019, with an IRR of 0.35 (95% CI: 0.18–0.67).

Among children aged 1–2 years, mean annual incidence pre‐COVID was approximately half that in infants (1.08 ± 0.25 vs. 2.62 ± 1.29 per 1000 persons), with less interannual variation; incidence in 2022 was lower than any pre‐COVID year. From 3 years of age, mean annual incidence during the pre‐COVID baseline was lowest in the 6‐ to 14‐year age group (0.09 ± 0.03), and in young adults aged 15–29 years (0.13 ± 0.06), increasing steadily with age thereafter to reach 1.19 ± 0.57 in adults ≥75 years, among whom interannual variation was also seen but was less marked than younger age groups.

**FIGURE 1:**
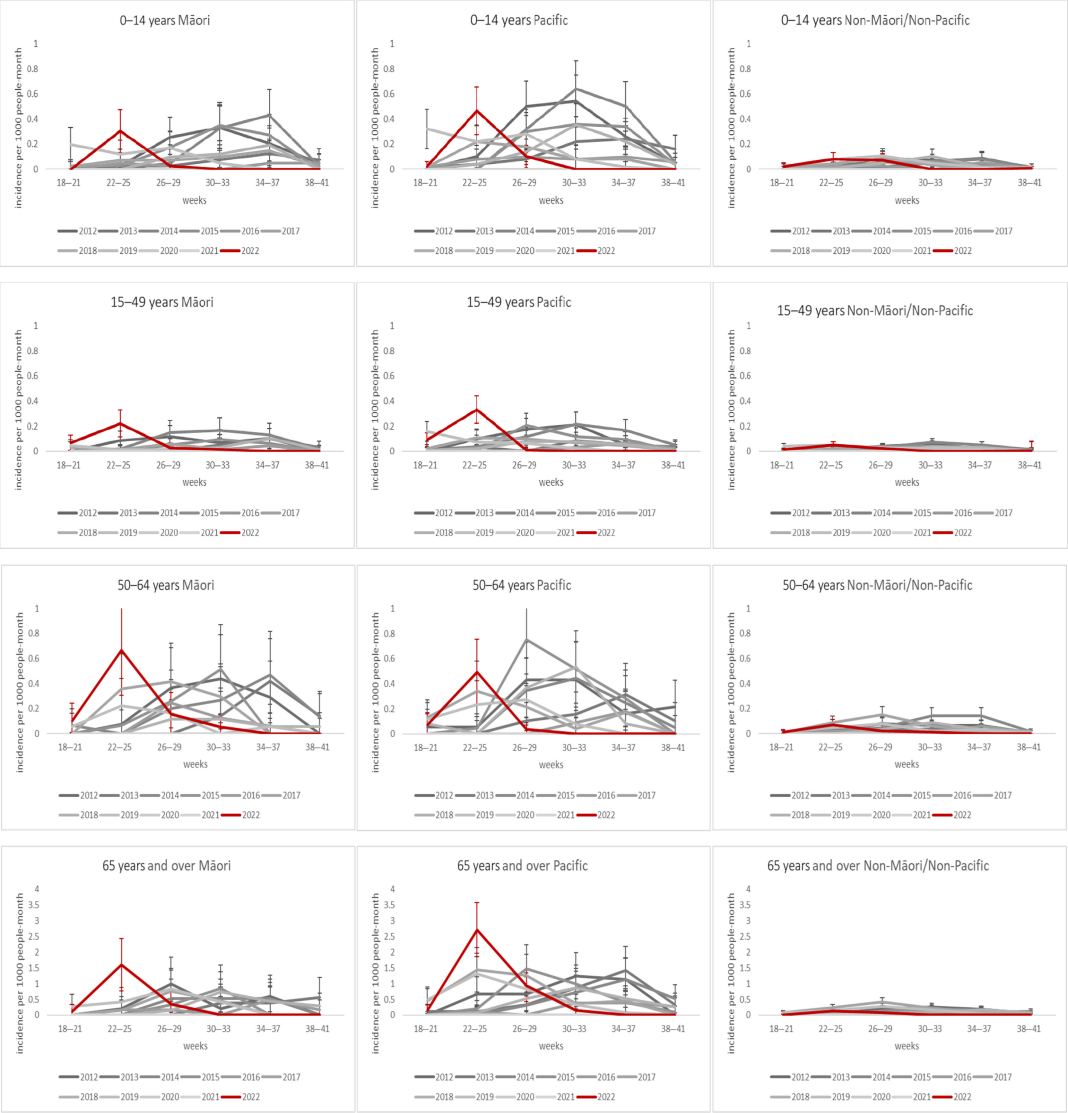
Influenza incidence by age and ethnic group for 2012–2022.

In all age groups over 3 years, annual incidence in 2022 was similar to mean annual incidence pre‐COVID except for adults ≥75 years, among whom annual incidence was significantly lower than mean pre‐COVID (IRR 0.66; 95% CI: 0.44–0.99). However, clear differences in age‐specific incidence were apparent between the three ethnic groups (Māori, Pacific and NMNP). Annual (Table 2) and monthly (Figure 1) incidences were higher among Māori and Pacific people than NMNP, across all age groups during pre‐COVID years and in 2022. Patterns of peak monthly incidence varied by age and ethnicity in 2022. In people of all ethnicities less than 15 years of age, peak monthly incidence in 2022 occurred earlier than any previous year, but peaks were not higher than baseline. In contrast, among people 15 years and over, Māori and Pacific had higher peak monthly incidence of shorter duration in 2022 than in any pre‐COVID year, significantly higher in Pacific people 15–49 years, whereas NMNP people had a peak monthly incidence in 2022, which was lower and of comparable duration to previous years (Figure 1).

**TABLE 2:** Annual incidence of influenza SARI by age and ethnic group, Auckland, New Zealand, 2012–2022.

Age group, years	Ethnic group	Year									
		2012	2013	2014	2015	2016	2017	2018	2019	2012–2019	2022
≤14	Māori	0.87 (0.60–1.22)	0.33 (0.18–0.57)	0.83 (0.57–1.17)	0.85 (0.59–1.19)	0.17 (0.07–0.36)	0.44 (0.26–0.70)	0.41 (0.24–0.66)	0.54 (0.34–0.82)	0.55 (0.34–0.83)	0.33 (0.17–0.56)
	Pacific	1.45 (1.14–1.83)	0.76 (0.54–1.05)	**1.52** ^**a**^ **(1.20–1.91)**	1.04 (0.78–1.36)	0.44 (0.27–0.66)	0.58 (0.39–0.83)	0.79 (0.57–1.08)	0.92 (0.68–1.23)	0.93 (0.68–1.24)	0.59 (0.39–0.85)
	NMNP	0.21 (0.14–0.32)	0.19 (0.11–0.28)	0.17 (0.10–0.26)	0.18 (0.11–0.28)	0.11 (0.06–0.19)	0.21 (0.13–0.31)	0.20 (0.13–0.30)	0.28 (0.19–0.39)	0.19 (0.12–0.29)	0.19 (0.12–0.28)
15–49	Māori	0.37 (0.23–0.56)	0.20 (0.10–0.35)	0.48 (0.32–0.68)	0.21 (0.11–0.36)	0.08 (0.03–0.18)	0.14 (0.06–0.26)	0.16 (0.08–0.29)	0.09 (0.03–0.19)	0.21 (0.11–0.35)	0.33 (0.21–0.50)
	Pacific	0.54 (0.40–0.73)	0.22 (0.13–0.35)	0.58 (0.43–0.77)	0.44 (0.31–0.60)	0.14 (0.07–0.24)	0.35 (0.24–0.50)	0.25 (0.16–0.37)	0.31 (0.21–0.45)	0.35 (0.23–0.49)	0.43 (0.31–0.58)
	NMNP	0.14 (0.10–0.18)	0.10 (0.06–0.13)	**0.18** ^**a**^ **(0.14–0.23)**	0.11 (0.08–0.15)	0.05 (0.03–0.08)	0.08 (0.06–0.12)	0.08 (0.06–0.11)	0.17 (0.13–0.21)	0.11 (0.08–0.15)	0.09 (0.06–0.13)
50–64	Māori	1.17 (0.67–1.90)	0.70 (0.34–1.30)	1.14 (0.67–1.83)	0.84 (0.45–1.43)	0.43 (0.17–0.89)	1.07 (0.63–1.69)	0.34 (0.12–0.74)	0.50 (0.23–0.95)	0.75 (0.39–1.32)	0.98 (0.59–1.53)
	Pacific	1.35 (0.87–1.20)	0.68 (0.36–1.16)	1.09 (0.68–1.65)	**1.60** ^**a**^ **(1.11–2.24)**	0.27 (0.10–0.58)	0.90 (0.56–1.38)	1.07 (0.70–1.56)	0.71 (0.42–1.12)	0.94 (0.58–1.45)	0.60 (0.35–0.96)
	NMNP	0.24 (0.17–0.35)	0.14 (0.08–0.22)	**0.36** ^**a**^ **(0.26–0.48)**	0.13 (0.08–0.21)	0.11 (0.06–0.18)	**0.33** ^**a**^ **(0.24–0.44)**	0.18 (0.11–0.26)	0.19 (0.13–0.28)	0.20 (0.13–0.30)	0.12 (0.07–0.19)
≥65	Māori	1.97 (0.95–3.63)	1.32 (0.53–2.72)	1.61 (0.73–3.05)	1.19 (0.48–2.44)	0.97 (0.35–2.10)	1.38 (0.63–2.63)	1.62 (0.81–2.89)	1.92 (1.05–3.23)	1.50 (0.68–2.83)	2.06 (1.22–3.26)
	Pacific	3.74 (2.57–5.25)	3.17 (2.12–4.55)	2.70 (1.76–3.95)	3.16 (2.16–4.47)	**1.13** ^**b**^ **(0.59–1.98)**	3.98 (2.89–5.34)	2.25 (1.47–3.30)	3.05 (2.15–4.21)	2.87 (1.94–4.11)	3.94 (2.97–5.13)
	NMNP	**0.73** ^**a**^ **(0.56–0.93)**	0.39 (0.27–0.55)	0.39 (0.27–0.55)	**0.50** ^**a**^ **(0.37–0.67)**	0.30 (0.20–0.43)	**1.05** ^**a**^ **(0.86–1.28)**	0.38 (0.27–0.52)	**0.73** ^**a**^ **(0.57–0.91)**	0.56 (0.42–0.73)	0.22 (0.14–0.32)

*Note*: All values are incidence per 1000 persons (95% confidence interval). No cases of influenza‐associated SARI were detected in 2020 and 2021. Bold values indicate incidence and CI significantly above or below that in 2022.

Abbreviation: NMNP, non‐Māori/non‐Pacific.

^a^ Incidence and CI above that in 2022.

^b^ Incidence and CI below that in 2022.

Annual incidence in 2022 was higher in younger (15–49 years) and older (≥65 years) Māori and Pacific than NMNP adults, IRR (95% CI) among Māori 1.58 (0.80–3.08) and Pacific 1.23 (0.77–1.94) aged 15–49 years and 1.37 (0.62–3.05) for Māori and 1.37 (0.87–2.13) for Pacific adults ≥65 years compared with NMNP. Pacific adults ≥65 years had the highest annual incidence of all ethnicities when age was grouped as 0–14, 15–49, 50–65 and 65+, across all years, with 2022 higher than almost all years in the pre‐COVID baseline. In contrast, among NMNP adults ≥65 years, annual and peak monthly incidences in 2022 were significantly lower than the pre‐COVID baseline, with an IRR of 0.38 (0.24–0.62).

## 4 Discussion

Following the large reduction in influenza circulation across most of the world during 2020 and 2021, there was concern about increased incidence and severity following reintroduction, due to a decline in population immunity. There are limited data on age or ethnicity during the 2022 influenza rebound [5, 17, 18, 19, 20]. In Aotearoa/NZ, significant long‐term disparities in the burden of severe respiratory viral infections for Māori and Pacific ethnic groups have been previously documented [9], likely due to crowded and inferior housing and other indicators of socioeconomic deprivation. Accordingly, it was important to examine severe influenza by age and ethnic group following the reintroduction of influenza in 2022, with valid comparison with the pre‐COVID era made possible by unique long‐term baseline data on SARI cases in Auckland, NZ.

Our findings suggest that children were not disproportionately affected by the influenza resurgence in 2022 in NZ. In infants, although incidence was higher than in other age groups, both pre‐COVID and in 2022, it was lower in 2022 than in most pre‐COVID years. This suggests that, despite the absence of influenza transmission for the previous 2 years, maternally acquired immunity to influenza among infants was not substantially different in 2022, although it is also possible that parental practices with respect to exposure of infants to persons with respiratory symptoms may have also contributed. In children aged 3–14 years, incidence in 2022 was also similar to pre‐COVID years, again suggesting the lack of a substantial immunity gap. Among all children aged 0–14 years, we found no differences in incidence patterns by ethnicity in 2022. Although incidence was higher among Māori and Pacific children during the pre‐COVID baseline, peak monthly incidence in 2022 was similar to the pre‐COVID baseline in all ethnic groups.

In contrast, we found very different patterns among Māori and Pacific adults ≥65 years during the 2022 resurgence, with SARI‐influenza incidence higher than almost all pre‐COVID years, whereas among NMNP adults ≥65 years, SARI‐influenza in 2022 was strikingly low and lower than all pre‐COVID years. A number of factors may have contributed to this marked difference in hospitalized influenza. Influenza vaccination coverage was 10–15% lower between 2020 and 2021 among Māori adults over 65 years who also had a higher prevalence of comorbidities than among non‐Māori, non‐Pacific adults [21]. Although we did not have data on influenza vaccination status in influenza‐SARI cases over 65 years of age, it seems unlikely that differences in vaccine coverage and prevalence of comorbidities alone could result in the approximately tenfold higher incidence of influenza‐SARI in 2022 among Māori and Pacific adults compared with NMNP. We consider it likely that a more prominent contributor to this large discrepancy is the much higher proportion of Māori and Pacific adults over 65 years residing in crowded households with children (9.2% and 27.4%, respectively) than NMNP adults (2.7%), with crowding more marked in Auckland, the location of our study than other areas of New Zealand [22]. In addition, NMNP households including adults >65 years are likely to have had greater capacity to self‐isolate in 2022, especially during the first half of 2022 when COVID‐19 when a high level of public health and social measures were still in place.

This analysis had some limitations, including the lack of data on influenza‐positive cases that did not meet the SARI case definition and confounding variables for likelihood of influenza hospitalization such as influenza vaccination and comorbidity status. However, the magnitude of the increased incidence we documented among older Māori and Pacific adults in the Auckland region of Aotearoa/NZ is unlikely to be accounted for solely by vaccination and comorbidity, and therefore, other factors such as household crowding may be relevant. We have relied on confidence intervals to detect noteworthy differences in rates, and this may lead to underestimating significant, smaller differences in rates.

In summary, our analysis has documented that, based on a valid and consistent pre‐COVID baseline, even in a setting where influenza was completely absent for 2 years with almost no community transmission of SARS‐CoV‐2, overall annual incidence of SARI‐influenza hospitalization did not change, although this does not capture short‐term hospital burden due to sharp but time‐limited increases in cases [5]. In 2022, patterns of SARI‐influenza were similar to prepandemic patterns in children of all ethnicities, but among adults over 65 years, we documented substantial excess hospitalizations in Māori and Pacific adults, who predominantly live in less advantaged areas of Auckland. These findings highlight the importance of more detailed examination of patterns of influenza activity post‐COVID, as these differences by age and ethnicity were concealed in the whole of population analysis and have important implications for prevention.

## Author Contributions

**Isabella Cheung MY:** investigation, writing – original draft, formal analysis, writing – review and editing. **Janine Paynter:** methodology, writing – review and editing, formal analysis, visualization, supervision. **David Broderick:** conceptualization, methodology, writing – review and editing, data curation. **Adrian Trenholme:** writing – review and editing, conceptualization. **Cass Byrnes A:** conceptualization, writing – review and editing. **Cameron Grant C:** conceptualization, writing – review and editing, supervision. **Qiu Huang S:** conceptualization, writing – review and editing. **Nikki Turner:** conceptualization, funding acquisition, writing – review and editing, supervision. **Peter McIntyre:** conceptualization, investigation, writing – review and editing, methodology, funding acquisition, supervision.

## 5 Conflicts of Interest

Janine Paynter has conducted other research work for which her employer received funding from GSK. Her employer has also received funds for advisory board membership and presentations she has done on behalf of CSL Seqirus. Nikki Turner is the medical director for the Immunisation Advisory Centre, which holds a contract with the government agency, Health New Zealand, to support the delivery of the annual national influenza vaccination campaign. Sue Huang has conducted other research work for which her employer received funding from Icosavax. Her employer has also received funds for advisory board membership and presentations she has done on behalf of CSL Seqirus. Adrian Trenholme is a general pediatrician working for Te Whatu Ora at Kidz First Hospital, South Auckland, and senior lecturer with the University of Auckland. In the past, Peter McIntyre has worked as the Kidz First site investigator for RSV related studies with Novavax and Medimmune. He currently has a grant from the Fisher and Paykel Healthcare Foundation to study Implicit Bias. All other authors declare they have no conflicts of interest.

### Peer Review

The peer review history for this article is available at https://www.webofscience.com/api/gateway/wos/peer‐review/10.1111/irv.70029.

## Data Availability

The data that support the findings of this study are available on request from the corresponding author. The data are not publicly available due to privacy or ethical restrictions.

## References

[1] J. R. Ortiz , V. Sotomayor , O. C. Uez , et al., “Strategy to Enhance Influenza Surveillance Worldwide,” Emerging Infectious Diseases 15, no. 8 (2009): 1271–1278, 10.3201/eid1508.081422. 19751590 PMC2815958

[2] Q. S. Huang , M. Baker , C. McArthur , et al., “Implementing Hospital‐Based Surveillance for Severe Acute Respiratory Infections Caused by Influenza and Other Respiratory Pathogens in new Zealand,” Western Pacific Surveillance and Response Journal: WPSAR 5, no. 2 (2014): 23–30, 10.5365/wpsar.2014.5.1.004. 25077034 PMC4113656

[3] Q. S. Huang , T. Wood , L. Jelley , et al., “Impact of the COVID‐19 Nonpharmaceutical Interventions on Influenza and Other Respiratory Viral Infections in new Zealand,” Nature Communications 12, no. 1 (2021): 1001, 10.1038/s41467-021-21157-9. PMC7881137 33579926

[4] F. Bonacina , P. Y. Boëlle , V. Colizza , O. Lopez , M. Thomas , and C. Poletto , “Global Patterns and Drivers of Influenza Decline During the COVID‐19 Pandemic,” International Journal of Infectious Diseases 128 (2023): 132–139, 10.1016/j.ijid.2022.12.042. 36608787 PMC9809002

[5] Q. S. Huang , N. Turner , T. Wood , et al., “Impact of the COVID‐19 Related Border Restrictions on Influenza and Other Common Respiratory Viral Infections in New Zealand,” Influenza and Other Respiratory Viruses 18, no. 2 (2024): e13247, 10.1111/irv.13247. 38350715 PMC10864123

[6] S. S. Lee , C. Viboud , and E. Petersen , “Understanding the Rebound of Influenza in the Post COVID‐19 Pandemic Period Holds Important Clues for Epidemiology and Control,” International Journal of Infectious Diseases 122 (2022): 1002–1004, 10.1016/j.ijid.2022.08.002. 35932966 PMC9349026

[7] S. D. Marbus , W. van der Hoek , J. T. van Dissel , and A. B. van Gageldonk‐Lafeber , “Experience of Establishing Severe Acute Respiratory Surveillance in the Netherlands: Evaluation and Challenges,” Public Health in Practice 1 (2020): 100014, 10.1016/j.puhip.2020.100014. 34171043 PMC7260511

[8] O. Eales , M. J. Plank , B. J. Cowling , et al., “Key Challenges for Respiratory Virus Surveillance While Transitioning out of Acute Phase of COVID‐19 Pandemic,” Emerging Infectious Diseases 30, no. 2 (2024): e230768, 10.3201/eid3002.230768. 38190760 PMC10826770

[9] M. G. Baker , L. T. Barnard , A. Kvalsvig , et al., “Increasing Incidence of Serious Infectious Diseases and Inequalities in New Zealand: A National Epidemiological Study,” Lancet 379, no. 9821 (2012): 1112–1119, 10.1016/S0140-6736(11)61780-7. 22353263

[10] N. Sheridan , T. Love , and T. Kenealy , “Primary Care Models Study Group. Is There Equity of Patient Health Outcomes Across Models of General Practice in Aotearoa New Zealand? A National Cross‐Sectional Study,” International Journal for Equity in Health 22, no. 1 (2023): 79, 10.1186/s12939-023-01893-8. 37143152 PMC10157126

[11] J. Atkinson , C. Salmond , and P. Crampton , NZDep2013 Index of Deprivation (Wellington: University of Otago, 2014, accessed 17 February 2024) https://www.otago.ac.nz/__data/assets/pdf_file/0029/318458/nzdep2013‐index‐of‐deprivation‐research‐report‐069936.pdf.

[12] D. C. Mare , P. Mawson , and J. Timmins , Deprivation in New Zealand: Regional Patterns and Changes (Wellington: The Treasury, 2001, accessed 17 February 2024) https://www.treasury.govt.nz/publications/wp/deprivation‐new‐zealand‐regional‐patterns‐and‐changes‐wp‐01‐09.

[13] J. Gurney , J. Stanley , and D. Sarfati , “The Inequity of Morbidity: Disparities in the Prevalence of Morbidity Between Ethnic Groups in new Zealand,” Journal of Comorbidity 10 (2020): 2235042X20971168, 10.1177/2235042X20971168. PMC7658519 33224894

[14] Q. S. Huang , N. Turner , M. G. Baker , et al., “Southern Hemisphere Influenza and Vaccine Effectiveness Research and Surveillance,” Influenza and Other Respiratory Viruses 9, no. 4 (2015): 179–190, 10.1111/irv.12315. 25912617 PMC4474494

[15] “New Zealand Period Life Tables: 2012–14,” Stats NZ [Internet], accessed 16 January 2024, (2015), https://www.stats.govt.nz/information‐releases/new‐zealand‐period‐life‐tables‐201214/.

[16] P. A. Harris , R. Taylor , R. Thielke , J. Payne , N. Gonzalez , and J. G. Conde , “Research Electronic Data Capture (REDCap)—A Metadata‐Driven Methodology and Workflow Process for Providing Translational Research Informatics Support,” Journal of Biomedical Informatics 42, no. 2 (2009): 377–381, 10.1016/j.jbi.2008.08.010. 18929686 PMC2700030

[17] C. M. Thomas , E. B. White , N. Kojima , et al., “Early and Increased Influenza Activity Among Children—Tennessee, 2022–23 Influenza Season,” MMWR. Morbidity and Mortality Weekly Report 72, no. 3 (2023): 49–54, 10.15585/mmwr.mm7203a1. 36656786 PMC9869745

[18] E. B. White , A. O'Halloran , D. Sundaresan , et al., “High Influenza Incidence and Disease Severity Among Children and Adolescents Aged <18 Years—United States, 2022–23 Season,” MMWR. Morbidity and Mortality Weekly Report 72, no. 41 (2023): 1108–1114, 10.15585/mmwr.mm7241a2. 37824430 PMC10578954

[19] S. L. Kwon and B. I. Kim , “How COVID‐19 Shifted the Seasonal Flu in Korea,” Influenza and Other Respiratory Viruses 17, no. 3 (2023): e13113, 10.1111/irv.13113. 36875206 PMC9978056

[20] M. J. Trent , A. Moa , and C. R. MacIntyre , ““I'll Be Back”: Australia's Experience of Flu in 2022,” BMJ 379 (2022): o2998, 10.1136/bmj.o2998. 36517068

[21] N. Wehipeihana , K. Sebire , K. Spee , and J. Oakden , In Pursuit of Māori Health Equity. Evaluation of the Māori Influenza and Measles Vaccination Programme (Ministry of Health: Wellington, 2022. accessed 16 January 2024) https://www.health.govt.nz/publication/evaluation‐maori‐influenza‐and‐measles‐vaccination‐programme‐pursuit‐maori‐health‐equity.

[22] “Almost 1 in 9 People Live in a Crowded House,” Stats NZ [Internet], (2020), https://www.stats.govt.nz/news/almost‐1‐in‐9‐people‐live‐in‐a‐crowded‐house.

